# Conditional Deletion of *Pten* Leads to Defects in Nerve Innervation and Neuronal Survival in Inner Ear Development

**DOI:** 10.1371/journal.pone.0055609

**Published:** 2013-02-05

**Authors:** Hyung Jin Kim, Hae-Mi Woo, Jihee Ryu, Jinwoong Bok, Jin Woo Kim, Sang Back Choi, Mi-Hyun Park, Hyun-Young Park, Soo Kyung Koo

**Affiliations:** 1 Center for Biomedical Sciences, National Institute of Health, Osong Health Technology Administration Complex 643, Yeonje-ri, Osong-eup, Cheongwon-gun, Chungcheongbuk-do, South Korea; 2 Department of Anatomy, BK21 Project for Medical Science, Yonsei University College of Medicine, Seoul, South Korea; 3 Department of Biological Sciences, Korea Advanced Institute of Science and Technology (KAIST), 291 Daehak-ro, Yuseong-gu, Daejeon, South Korea; Universitat Pompeu Fabra, Spain

## Abstract

All cellular phenomena and developmental events, including inner ear development, are modulated through harmonized signaling networks. Phosphatase and tensin homolog deleted on chromosome 10 (PTEN), a tumor suppressor, is a major signaling component involved in cross talk with key regulators of development; *i*.*e*., Wnt, Notch, and bone morphogenetic proteins. Although *Pten* function has been studied in various systems, its role in inner ear development is poorly understood. Here, we used inner ear-specific *Pten* conditional knockout mice and examined the characteristics of the inner ear. In a detailed analysis of the phenotype, reduced cochlear turning and widened epithelia were observed. Phalloidin staining of sensory epithelium revealed that hair cell patterns were disturbed; *i*.*e*., additional rows of hair cells were discovered. The neural abnormality revealed a reduction in and disorganization of nerve fibers, including apoptosis at the neural precursor stage. *Pten* deficiency induced increased phosphorylation of Akt at Ser473. The elevation of inhibitory ***g***lycogen synthase kinase 3β Ser9 phosphorylation (pGSK3β) was sustained until the neuronal differentiation stage at embryonic day 14.5, instead of pGSK3β downregulation. This is the first report on the influence of Pten/Akt/GSK3β signaling on the development of spiral ganglia. These results suggest that *Pten* is required for the maintenance of neuroblast number, neural precursors, and differentiation in the inner ear.

## Introduction

Early in auditory system development, the otic placode undergoes invagination to form otocysts, which generate a complex membranous labyrinth and ganglia through a series of morphological events via harmonized coordination of various signaling molecules such as fibroblast growth factors, Notch, bone morphogenetic proteins, and Wnt [1], [2], [3], [4], [5]. To form the cochleovestibular ganglion (CVG) complex, neuroblasts delaminate from otic epithelium into the adjacent mesoderm between embryonic day (E)9.5 and E11.5 [6], [7]. After delamination, the CVG complexes are separated into the spiral and the vestibular ganglia. Spiral ganglia undergo maturation processes around E11.5 to E15.5 (with a peak at E13.5) in a basal-to-apical innervation pattern [7]. The neurites of the spiral ganglia extend toward the developing sensory epithelium (*i*.*e*., Kölliker’s organ) under the guidance of neurotrophins such as brain-derived neurotrophic factor and neurotrophin-3 (NT-3) [8]. Because development of CVG neurons in the inner ear is complicated, involving a variety of developmentally regulated signaling pathways, the molecular mechanisms of spiral ganglia differentiation are not well understood.

PTEN is a key modulator of phosphatidylinositol 3-OH kinase (PI3K) signaling that involves the regulation of diverse cellular events such as growth, differentiation, and migration, and specialized developmental functions [9], [10], [11]. PTEN dephosphorylates phosphatidylinositol-3,4,5-triphosphate (PIP3) to produce phosphatidylinositol-4,5-biphosphate, thereby directly affecting the PI3K pathway [12]. The loss of PTEN leads to PI3K-hyperactivated accumulation of PIP3, which promotes Akt phosphorylation and induces phosphorylation of downstream effectors such as the mammalian target of rapamycin, forkhead box 1, and g*lycogen synthase kinase 3 (*GSK3) α/β [13], [14], [15]. Recently, *GSK3* has been reported to be a regulator of neurogenesis that coordinates multiple signaling pathways involving cell proliferation and differentiation of progenitors [16], [17]. During inner ear development, PI3K/Akt signaling has also been associated with insulin-like growth factor 1 (IGF-1). Mutation of the gene encoding its receptor (Igfr) is associated with severe cochlear defects and deafness [18], [19].

The function of *Pten* has been studied using transgenic animals. The conventional *Pten* knockout model exhibits early embryonic lethality, and hemizygous *Pten* mice undergo tumorigenesis in several organs [20], [21]. Conditional *Pten* deletion in neural precursors leads to a significantly enlarged brain, increased proliferative capacity, and neuronal hypertrophy [22], [23]. Haplo-insufficiency of *PTEN* in subventricular zone progenitor cells results in extensive migration and resistance to hydrogen peroxide-induced apoptosis [24]. Marino *et al*. [25] reported that loss of *Pten* disrupts cell layers in the developing cerebellum, suggesting a disturbance of migration and positioning. Treatment with *PTEN*-antisense oligonucleotide produces short neurites with apoptosis in the PC12 cell line [26]. These results indicate that *Pten* may participate in regulating neural organization, connection, and outgrowth, including neurodevelopment. In the inner ear, it has been reported that *Pten* expression is observed in the developing cochlea and that phosphorylated *Pten* accumulates in hair cell and supporting cell nuclei of the aging cochlea [27], [28]. Despite extensive studies of *Pten-*null phenotypes in various systems, little is known about the neuronal roles and mechanisms of *Pten* during inner ear development. In this study, we characterized the inner ear of a *Pten* conditional-knockout model, and showed that *Pten* is one of the key molecules in neuronal maintenance of the developing CVG population.

## Materials and Methods

### Ethics Statement

All animal procedures were conducted according to the guidelines for the use of laboratory animals and were approved by the Institutional Animal Care and Use Committee at the Korea Food and Drug Administration or Korea Centers for Disease Control and Prevention (10Sikyak30 and KCDC-017-11).

### Mouse Lines

*Pten^tm1Hwu^* (stock number 006440), Tg(Neurog1-cre/ESR_1_)_1_Good (stock number 008529), and Tg(Atoh1-cre/Esr_1_^*^)_14_Fsh (stock number 007684) mice were purchased from Jackson Laboratories (Bar Harbor, ME, USA) and Tg(Pax2-Cre)1Akg (stock number 010569-UNC) mice were purchased from MMRRC (UNC, Chapel Hill, NC, USA). Tg(Neurog1-cre/ESR_1_)_1_Good, Tg(Atoh1-cre/Esr_1_^*^)_14_Fsh, and Tg(Pax2-Cre)1Akg mice were mated with *Pten^tm1Hwu^* mice to generate *Pax2^Cre/+^*;*Pten^loxP/+^*, *Neurog1^Cre/+^*;*Pten^loxP/+^*, and *Atoh^Cre/+^*;*Pten^loxP/+^* double-transgenic animals. These animals were crossed with *Pten^tm1Hwu^* mice to generate conditional knockout mice of four genotypes: *Cre^+/+^*;*Pten^loxP/+^*, *Cre^Cre/+^*;*Pten^loxP/+^*, *Cre^+/+^*;*Pten^loxP/loxP^*, and *Cre^Cre/+^*;*Pten^loxP/loxP^*. *Cre^Cre/+^*;*Pten^loxP/loxP^* mice were selected as cKO mice. Mice of the other two phenotypes (*Pten^loxP/+^* and *Pten^loxP/loxP^*) are referred to as “wild type” because they have no abnormal phenotype. The morning of the day of the vaginal plug was considered E0.5. Embryos were harvested between E10.5 and E18.5 for the experiments. Genotyping was performed using the following PCR primers: *PtenloxP*-Fwd, 5′-CTCCTCTACTCCATTCTTCCC-3′; *PtenloxP*-Rev, 5′-ACTCCCACCAATGAACAAAC-3′; *Cre*-Fwd, 5′-TGCATGATCTCCGGTATTGA-3′; and *Cre*-Rev, 5′-ATGGATTTCCGTCTCTGGTG-3′.

### Tamoxifen Injections

To induce the conditional knockout of *Pten* in *Neurog1^Cre/+^*;*Pten^loxP/loxP^* and *Atoh^Cre/+^*;*Pten^loxP/loxP^* mice, tamoxifen (Sigma-Aldrich, St. Louis, MO, USA) was administered by intraperitoneal (IP) injection, as described previously [29]. To minimize the abortion rate due to multiple daily injections, we diluted the tamoxifen to 10 mg/ml in corn oil and included progesterone at half the amount of tamoxifen. One dose of 1 mg per 40 g pregnant body weight was injected with a syringe and a 26-gauge needle.

### Paint-fill Analysis

Paint-fill analyses of the inner ears were performed as previously described [30]. The inner and outer lengths of paint-filled cochleae at E17.5 were calculated with ImageJ (NIH, Bethesda, MD, USA).

### Immunofluorescence

Embryos were harvested and fixed in 4% paraformaldehyde (PFA) at 4°C overnight, cryo-protected in 30% sucrose, embedded in OCT compound, and sectioned to a thickness of 10 µm using a cryostat. The following primary antibodies were used: anti-PTEN mouse monoclonal (560002, 1∶20; BD Biosciences, Sparks, MD, USA), anti-NeuroD goat polyclonal (sc-1084, 1∶50; Santa Cruz Biotechnology, Santa Cruz, CA, USA), anti-Islet1 mouse monoclonal (40.2D6, 1∶50; Developmental Studies Hybridoma Bank, Iowa City, IA, USA), anti-Tuj1 mouse monoclonal (MMS-435P, 1∶400; Covance, Emeryville, CA, USA), anti-Tuj1 rabbit monoclonal (MRB-435P, 1∶400; Covance), anti-TrkC goat monoclonal (AF1404, 1∶25; R&D Systems, Minneapolis, MN, USA), anti-phospho-Akt rabbit monoclonal (4060, 1∶50; Cell Signaling Technology, Danvers, MA, USA), anti-phospho-GSK3β rabbit monoclonal (9323, 1∶50; Cell Signaling Technology), anti-cleaved caspase-3 rabbit monoclonal (9664, 1∶200; Cell Signaling Technology), anti-PCNA mouse monoclonal (ab29, 1∶1000, Abcam, Cambridge, MS, USA), fluorescein phalloidin (F432, 1∶200; Molecular Probes, Carlsbad, CA, USA), anti-Myosin VIIa rabbit polyclonal (ab3481, 1∶100; Abcam), anti-p75^NTR^ rabbit polyclonal (AB1554, 1∶100; Millipore, Billerica, MA, USA), anti-acetylated tubulin mouse monoclonal (T6793, 1∶500; Sigma-Aldrich), anti-Prox1 rabbit polyclonal (PRB-238C, 1∶2000; Covance), and anti-neurofilament rabbit monoclonal (N4142, 1∶100; Sigma-Aldrich) antibodies. After antigen retrieval with 10 mM citrate buffer for 20 min at 95°C, the sections were incubated in blocking solution and 10% normal donkey serum or 10% normal goat serum (Abcam) in phosphate-buffered saline with 0.2% Triton X-100 for 1 h prior to incubation with primary antibodies at 4°C overnight. Secondary fluorescent staining with Alexa Fluor 488 or Alexa Fluor 594 (1∶400; Molecular Probes) was performed at room temperature for 1 h. For whole-mount immunofluorescence, dissected cochleae were fixed with 4% PFA before further blocking and antibody labeling, as described above (without the antigen retrieval step). Fluorescence images were obtained with a confocal microscope (Carl Zeiss, Göttingen, Germany) or a fluorescence microscope (Axio Imager A1; Carl Zeiss).

### Neural Tracer Application

Nerve fibers were traced using NeuroVue® Red Plus (Polysciences, Warrington, PA, USA) to reveal the general pattern of innervation, as described previously [31], [32]. Briefly, a dye-coated filter triangle was used to cut the eighth cranial (vestibulocochlear) nerve in E17.5 or E18.5 *Pax2^Cre/+^*;*Pten^loxP/loxP^*, *Neurog1^Cre/+^*;*Pten^loxP/loxP^*, and *Atoh1^Cre/+^*;*Pten^loxP/loxP^* mice and their wild-type littermates (n>6 for each genotype). During diffusion, brains were incubated continuously in 4% PFA in 0.1 M phosphate buffer. After diffusion for 7 days at 37°C, the ears and otic ganglia were dissected away from the adjacent mesenchyme and photographed as whole mounts using an MZ16FA rhodamine filter set (Leica Microsystems, Wetzlar, Germany).

### Western Blotting Analysis

E14.5 inner ears from four wild-type and *Pax2^Cre/+^*;*Pten^loxP/loxP^* embryos were dissected, pooled, and homogenized in lysis buffer (Cell Signaling Technology). Approximately 10 µg of protein was separated by 10% SDS-PAGE and transferred onto a polyvinylidene fluoride membrane (Millipore). After blockade of nonspecific binding, the membranes were incubated with primary antibody and then incubated with the appropriate horseradish peroxidase-conjugated secondary antibody. The following primary antibodies were used: anti-PTEN rabbit monoclonal (9188, 1∶500, Cell Signaling Technology), anti-phospho-Akt (Ser473) rabbit monoclonal (4060, 1∶500, Cell Signaling Technology), anti-Akt rabbit monoclonal (4691, 1∶500, Cell Signaling Technology), anti-phospho-GSK3β rabbit monoclonal (Ser9) (9323, 1∶500, Cell Signaling Technology), anti-GSK3β rabbit monoclonal (9315, 1∶500, Cell Signaling Technology), and anti-β-actin (sc-47778, 1∶1000; Santa Cruz Biotechnology) antibodies. The signals in the Western blots were detected with an ECL kit (Thermo Scientific, Rockford, IL, USA). Band intensity ratios were calculated with ImageJ (NIH).

### Quantification of Cells

Neuroblasts were counted in 10-µm frozen sections. Consecutive sections through the entire CVG complex and epithelium were stained with anti-NeuroD and anti-Islet1 antibodies, and signal-positive cells with a clear nucleus were counted in every other section (every 20 µm) at 200× magnification using an Axio Imager A1 microscope (Carl Zeiss). Five to eight cochleae from four embryos of each genotype at E10.5 were analyzed. At E11.5, cleaved caspase-3-positive cells in the CVG were counted in every other section (every 20 µm) from three cochleae from three embryos. At E16.5, vestibular ganglia were enumerated in every fourth section (every 40 µm) from seven vestibules from four embryos. At E18.5, spiral ganglia were enumerated in every third section (every 30 µm) from three cochleae from three embryos.

### Statistical Analysis

Data are presented as means ± SEM. The statistical significance of differences was determined by unpaired Student’s *t*-test. Asterisks indicate level of significance (*P<0.05, **P<0.01, and ***P<0.001).

## Results

### Pten Expression Patterns during Inner Ear Development

**Figure 1:**
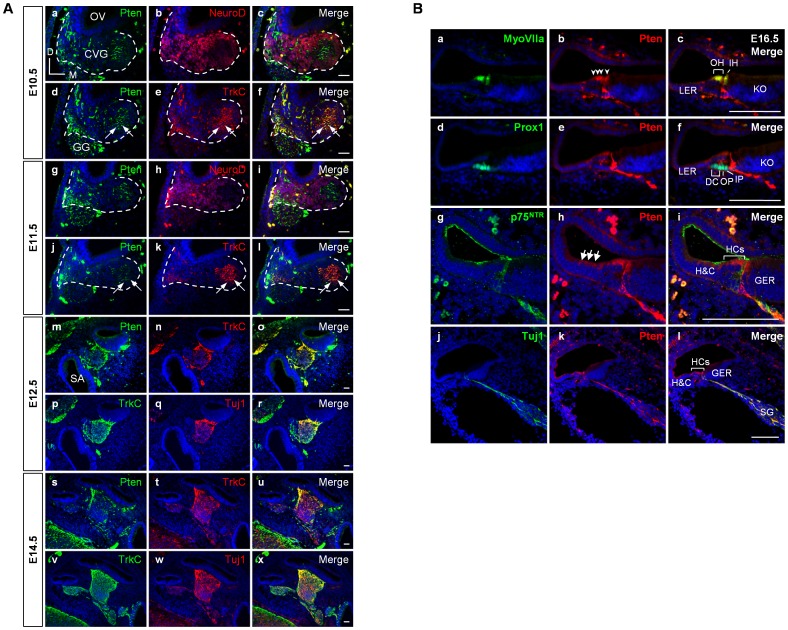
Pten expression patterns during inner ear development. (A, B) Pten expression in the inner ear was examined by immunofluorescence at E10.5, E11.5, E12.5, E14.5, and E16.5. (A) Pten protein (green) was first detected between E10.5 and E11.5 in the cytoplasm of the cochleovestibular ganglion (CVG) complex (white outlines in a–l), and mainly overlapped with TrkC-positive neural precursor cells (red) rather than co-localizing with NeuroD (red) (arrows in a–l). From E12.5 to E14.5, Pten (green) was clearly expressed in TrkC-positive cells (red) in cochlear ganglion neurons, which were defined by Tuj1-positive ganglia (red) (m–x). DAPI-stained nuclei (blue) are seen in all images. CVG, cochleovestibular ganglion; D, dorsal; GG, geniculate ganglion; M, medial; OV, otic vesicle; SA, saccule. Scale bars: 100 µm. (B) At E16.5, Pten-immunopositive signals (arrowheads in b) were observed in the MyoVIIa-positive hair cells (green) (a–c) but not in the Prox1-positive supporting cells (green) (d–f). Expression of Pten was detected in the Hensen’s and Claudius’ cells (arrows in h), Tuj1-positive neurons (green), but not in p75^NTR^-positive pillar cells (green) (g-i). DAPI-stained nuclei (blue) are seen in all images. DC, Deiter’s cell; GER, greater epithelial ridge; H&C, Hensen’s and Claudius’ cells; HCs, hair cells; IH, inner hair cell; IP, inner pillar cell; KO, Kölliker’s organ; LER, lesser epithelial ridge; OH, outer hair cell; OP, outer pillar cell; SG, spiral ganglion. Scale bars: 100 µm.

To evaluate the role of Pten during inner ear development, we examined the expression pattern of Pten from embryonic day (E)10.5 to E16.5 in the developing inner ear (Fig. 1). At E10.5, Pten was expressed in the CVG area but hardly detected in the inner ear epithelium (Fig. 1A, a–f). We compared Pten expression in the CVG with that of NeuroD, TrkC, and Tuj1. NeuroD is a marker of neuroblasts, and TrkC, a neurotrophin-3 receptor, is a marker for early differentiated neurons [33], [34]. At E10.5 to E11.5, most Pten expression in the CVG overlapped with TrkC signals rather than being co-expressed with NeuroD (arrows in Fig. 1A, f and l). From E12.5 to E14.5, during neuronal differentiation, Pten was clearly expressed in TrkC cochlear ganglia and pan-neuronal marker Tuj1-positive ganglion neurons (Fig. 1A, m–x). Pten immunoreactivity changed slightly over the course of inner ear development. During the early stages of development, Pten is expressed in the neuronal cell body since the outgrowth of neurites is not fully completed. From E16.5 to E18.5, Pten immunoreactivity remains in the neuronal cell body but seems to be stronger in the ends of neurites than at earlier stages (arrows in Fig. S1A). At E16.5, Pten-immunopositive signals were observed in the hair cells of the cochlear epithelium (arrowheads in Fig. 1B, b). In addition, Pten signals were detected in the area of Hensen’s and Claudius’ cells (arrows in Fig. 1B, h), which has not been reported previously. Tuj1-positive spiral ganglia showed Pten immunopositivity (Fig. 1B, j–l). Expression of Pten was also observed in the vestibular sensory epithelia, non-sensory epithelia, and ganglion (Fig. S1B).

### Characterization of the Inner ear in *Pax2^Cre/+^*;*Pten^loxP/loxP^* Embryos

**Figure 2:**
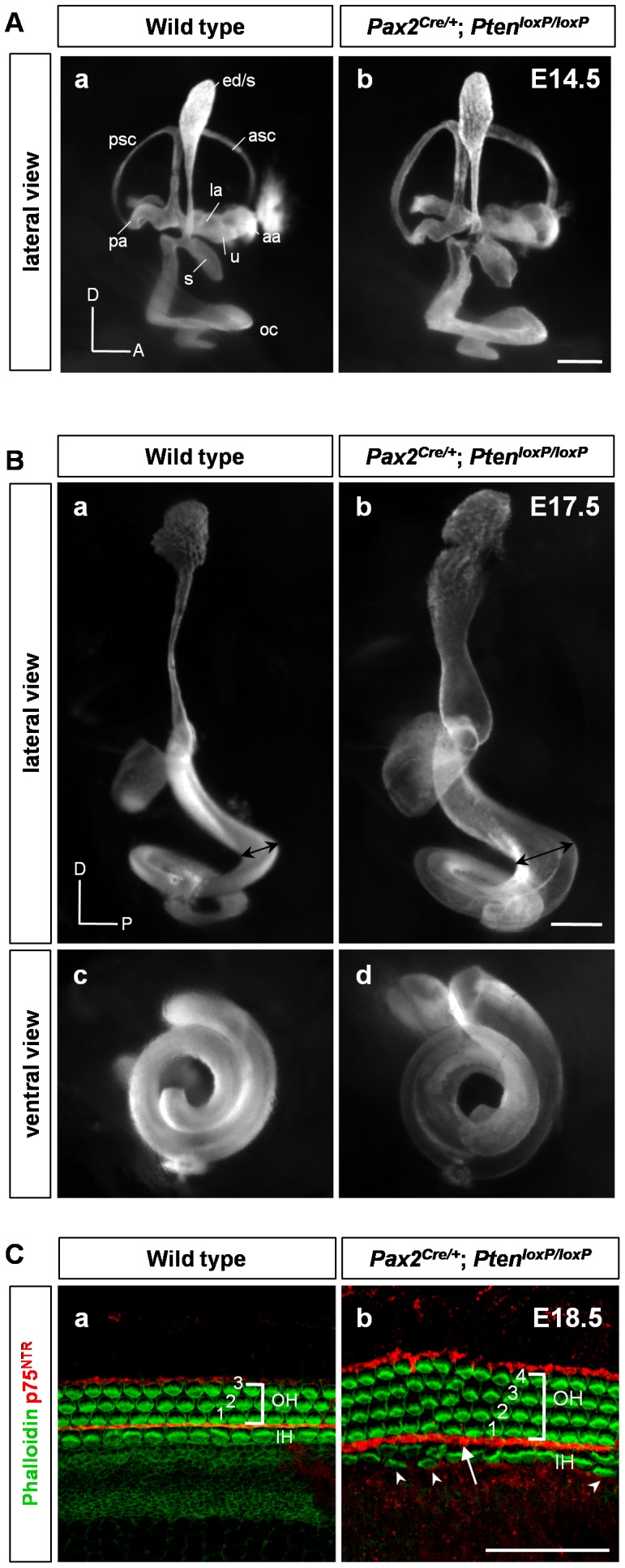
Inner ear phenotypes in *Pten* conditional knockout (cKO) mice. (A) Paint-filled inner ears of *Pten* cKO embryos at E14.5 shown in a lateral view. *Pten*-deficient inner ears showed abnormal morphology in the vestibule, including a thickened semicircular canal and canal pouches with a widened endolymphatic duct and common cruses. There was a grossly widened morphology with a coarse pattern in the cochlea. aa, anterior ampulla; asc, anterior semicircular canal; ed/s, endolymphatic duct and sac; la, lateral ampulla; oc, organ of Corti; pa, posterior ampulla; psc, posterior semicircular canal; s, saccule; u, utricle; A, anterior; D, dorsal. Scale bar: 50 µm. (B) At E17.5, the *Pten* deletion-induced morphology became more evident compared to that in the wild type (black double arrow in a, b). D, dorsal; P, posterior. Scale bar: 100 µm. (C) The morphological pattern of the epithelium was analyzed by whole-mount phalloidin with p75^NTR^ immunofluorescence. *Pten*-deficient mice showed additional rows of outer and inner hair cells, whereas wild-type mice showed three rows of outer hair cells and one row of inner hair cells at E18.5 (arrowheads in b). p75^NTR^-positive pillar cells were irregular and widened compared to those in wild-type mice (arrow in b). IH, inner hair cell; OH, outer hair cell. Scale bar: 100 µm.

To determine the gross morphology of the inner ear in *Pax2^Cre/+^*;*Pten^loxP/loxP^* (*Pten* conditional knockout (cKO)) embryos, we used the paint-fill technique with a total of four embryos at E14.5 and five embryos at E17.5 (Fig. 2). At E14.5, every component of the inner ear was identifiable but the membrane surface was coarse compared to that of the wild-type. In the vestibular part, the ears of *Pten* cKO embryos had thickened semicircular canals and canal pouches. The endolymphatic duct and common cruses were also wider. The *Pten*-deleted cochlea exhibited a grossly wide morphology with a coarse pattern but with slightly reduced turning at E14.5 (Fig. 2A, b). This widened morphology became more evident at E17.5 (Fig. 2B, b and d). Next, we measured the cochlea length at E17.5. The inner length of the *Pten*-deleted cochlea was decreased by 30% (69.6±6.2 in 4–6 cochleae, *P*<0.01) compared with that of wild-type cochlea, although no significant difference in outer length was observed (4–6 cochleae, *P*>0.95; data not shown). The coiling of the cochlea also changed slightly (Fig. 2B, d). In the wild type, the coiling of the cochlea reached 1.75 turns, but 9 of 10 cochleae in the affected ears had only 1.5 turns (Fig. 2B, c and d).

Next, we investigated cochlear sensory epithelium development and the organization of hair cells using fluorescent staining in whole-mount dissected sensory epithelium (Fig. 2C). In *Pten*-deleted embryos, rows of outer hair cells were staggered with an extra row of hair cells in the basal and mid turn, and the array of inner hair cells was sparsely disrupted by intruding extra inner hair cells (arrowheads in Fig. 2C, b), as assessed by phalloidin staining with p75^NTR^, a marker of inner pillar cells at E18.5. In contrast, numbers of hair cells in the vestibular sensory epithelium (*i*.*e*., utricle, saccule, and ampullae) did not show statistically significant deficits in *Pten* cKO embryos (data not shown). Although some irregular patterning was evident in the cochlear hair cell array, the general orientations of the hair cell bundles in *Pten* cKO embryos were different from those in wild-type mice (Fig. S2). The p75^NTR^-positive pillar cells were widened and occasionally missing (data not shown) compared to those of the wild type (arrow in Fig. 2C, b). These results indicated that *Pten* plays a role in the structural development of the organ of Corti.

### Neuronal Inner Ear Defects in the *Pten* cKO Embryos

**Figure 3:**
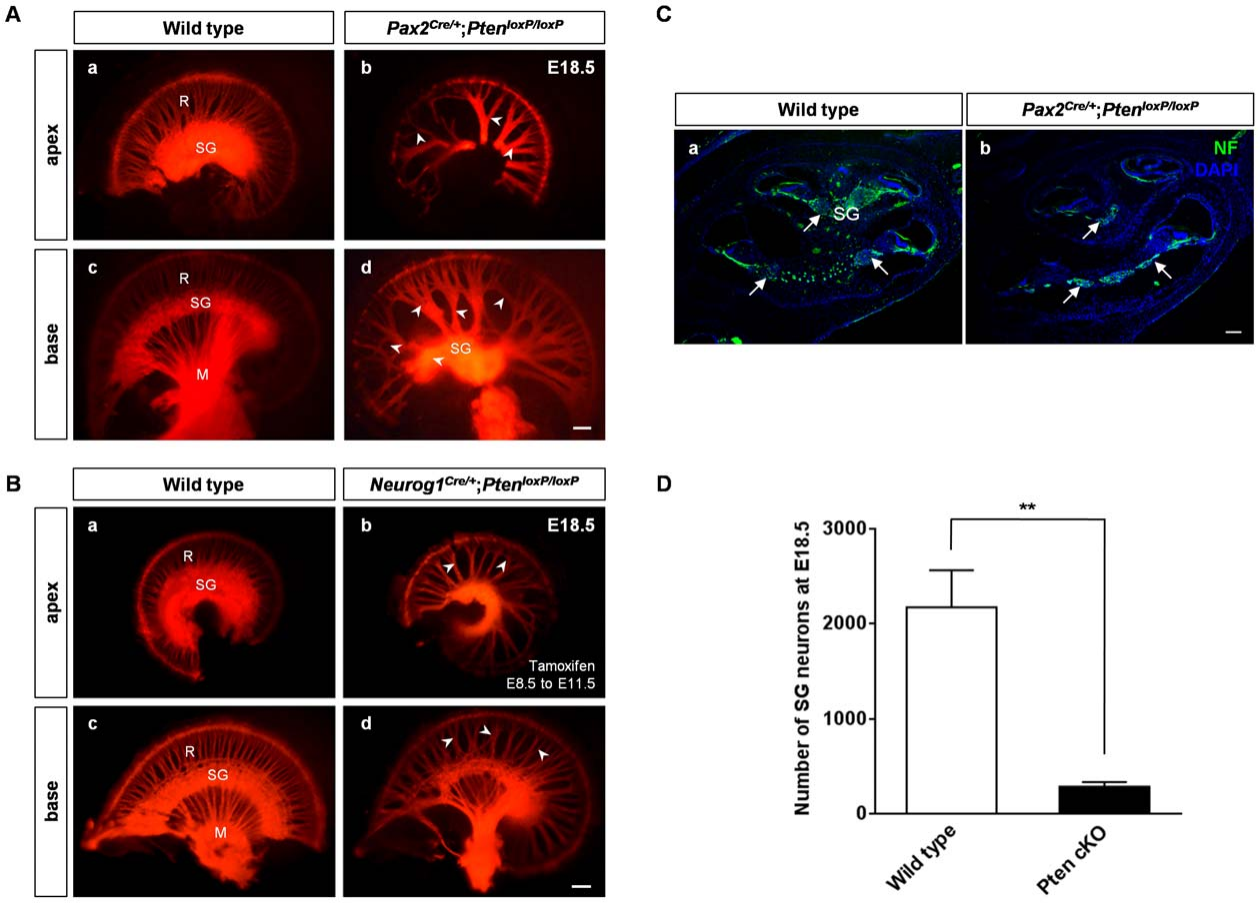
Cochlear innervation defects in *Pten-*deficient mice. (A, B) Patterns of cochlear innervation were assessed by NeuroVue-tracing at E18.5. (A) In *Pax2^Cre/+^*;*Pten^loxP/loxP^* mice, nerve innervation was evident in the apical and basal turns of the cochlea in wild-type (a, c) and *Pten* cKO (b, d) mice. *Pten*-deficient inner ears displayed sparse radial fibers, a disorganized pattern, and loss of spiral ganglia in the cochlea (arrowheads in b, d). Abnormal innervation was distributed evenly throughout the cochlea. M, modiolus; R, radial fibers; SG, spiral ganglion. Scale bar: 100 µm. (B) *Neurog1^Cre/+^*;*Pten^loxP/loxP^* mice were administered tamoxifen as an IP injection to perform tamoxifen-inducible deletion of *Pten* between E8.5 and E11.5. Neuronal abnormalities were seen in *Pax2^Cre/+^*;*Pten^loxP/loxP^* mice and similar innervation defects in *Neurog1^Cre/+^*;*Pten^loxP/loxP^* mice revealed spacing or several gathered radial fibers and a disorganized pattern in the innervation of the cochlea (arrowheads in b, d). The innervation defects were distributed evenly throughout the cochlea. M, modiolus; R, radial fibers; SG, spiral ganglion. Scale bar: 100 µm. (C, D) Neuronal loss in the spiral ganglion in *Pax2^Cre/+^*;*Pten^loxP/loxP^* mice at E18.5. (C) Neurofilament immunoreactivity (arrows) and (D) the number of the spiral ganglia were significantly decreased in *Pten* cKO mice compared to wild-type mice at E18.5 (3 cochleae, *P*<0.01). M, modiolus; R, radial fibers; SG, spiral ganglion. Scale bar: 100 µm.

We labeled the nerve innervation patterns by inserting a NeuroVue filter strip into the eighth nerve including both afferent and efferent nerves of inner ears at E18.5 to investigate neuronal phenotype (Fig. 3). The radial bundles of wild-type cochlea were evenly distributed (Fig. 3A, a and c). In contrast, *Pten* cKO mice had much thicker fascicles and showed possible pathfinding defects (arrowheads in Fig. 3A, b and d). Additionally, a shortened distance between the modiolus and spiral ganglion was observed in the basal turn, indicating an increased length between the spiral ganglion and hair cells from thirteen cochleae (201% increase, *P*<0.05; Fig. 3A, d). Ganglia in *Pax2^Cre/+^*;*Pten^loxP/loxP^* mice showed statistically significant percentage losses: 87% for spiral ganglion neurons at E18.5 (3 cochleae, *P*<0.01; arrows in Fig. 3C, D), and 58% for the vestibular ganglion at E16.5 (7 cochleae, *P*<0.01; Fig. S3).

Sensory neurons share a common progenitor with hair cells in the developing inner ear. Neural cell fate specification is decided by the expression of selector genes such as *Neurog1*, whereas hair cell fate is determined by Atoh1 [1], [6]. A ganglion defect can be caused by alteration of neurotrophic factors (*i*.*e*., NT-3 in the cochlea) secreted from the sensory domain of the epithelium [35]. Thus, sensory neuron and hair cell development may be influenced through cross-regulation. To verify the cause of the spiral ganglion defects, we performed selective deletion of *Pten* in either the sensory epithelium or the ganglia using *Atoh1^Cre/+^*;*Pten^loxP/loxP^* and *Neurog1^Cre/+^*;*Pten^loxP/loxP^* mice, respectively (Figs. S4 and 3B). When ganglion-selective deletion of *Pten* in the *Neurog1^Cre/+^*;*Pten^loxP/loxP^* mice was performed using tamoxifen injections from E8.5 to E11.5, badly disorganized ganglion innervation patterns resulted (arrowheads in Fig. 3B, b and d), whereas a normal innervation pattern was observed in the *Atoh1^Cre/+^*;*Pten^loxP/loxP^* mice (data not shown). Indeed, *Pten* depletion in *Neurog1^Cre/+^*;*Pten^loxP/loxP^* mice did not influence the hair cell pattern in the cochlear epithelia (Fig. S4A). Our data imply that the *Pten* cKO neuronal phenotype results from defects in progenitors of the *Neurog1*-positive neural lineage and did not influence sensory epithelial alteration.

### Loss of Neurons in the *Pten* cKO Spiral Ganglia

**Figure 4:**
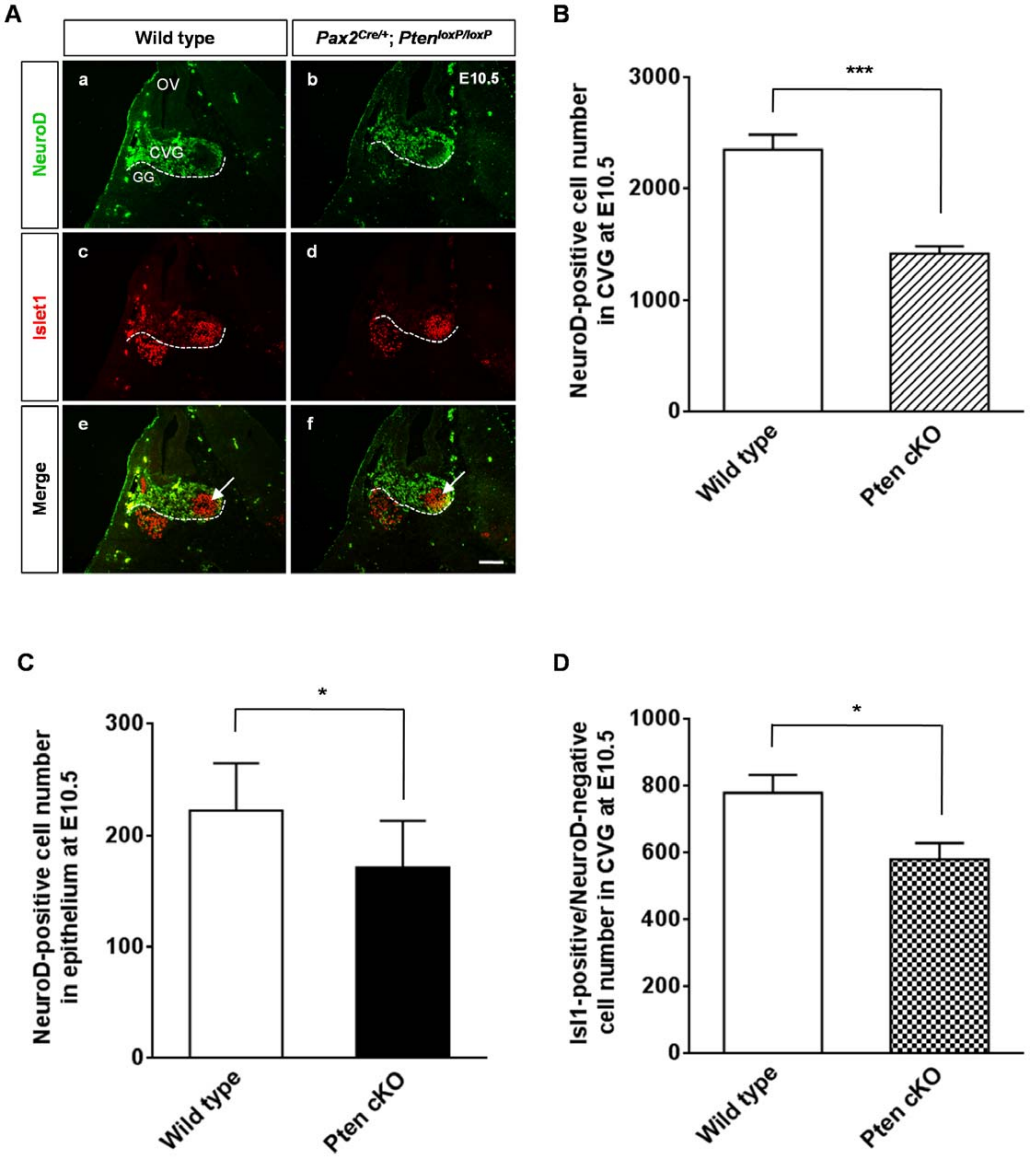
Reduction in neuronal cell number in the cochleovestibular ganglia (CVG) of *Pten* conditional knockout (cKO) mice. (A) NeuroD-positive cells are shown in green, and Islet1-positive cells are in red. Early matured neurons appeared to be expressed by both Islet1-positive and NeuroD-negative cells in the CVG at E10.5 (arrow in e, f). CVG, cochleovestibular ganglion; GG, geniculate ganglion; OV, otic vesicle. Scale bar: 100 µm. (B–D) Cell counts in the CVG (white outlines in A) and epithelium at E10.5. (B) The number of NeuroD-positive cells in *Pten*-deficient mice was reduced markedly to about half of that in wild-type mice (5 cochleae, *P*<0.001). (C) A significant reduction in NeuroD-positive cells in the inner ear epithelium was observed in *Pten* cKO mice (8 cochleae, *P*<0.05). (D) Early differentiated neurons expressed by Islet1-positive/NeuroD-negative in *Pten*-deficient mice showed a significant loss compared to those in wild-type mice (6 cochleae, *P*<0.05).

**Figure 5:**
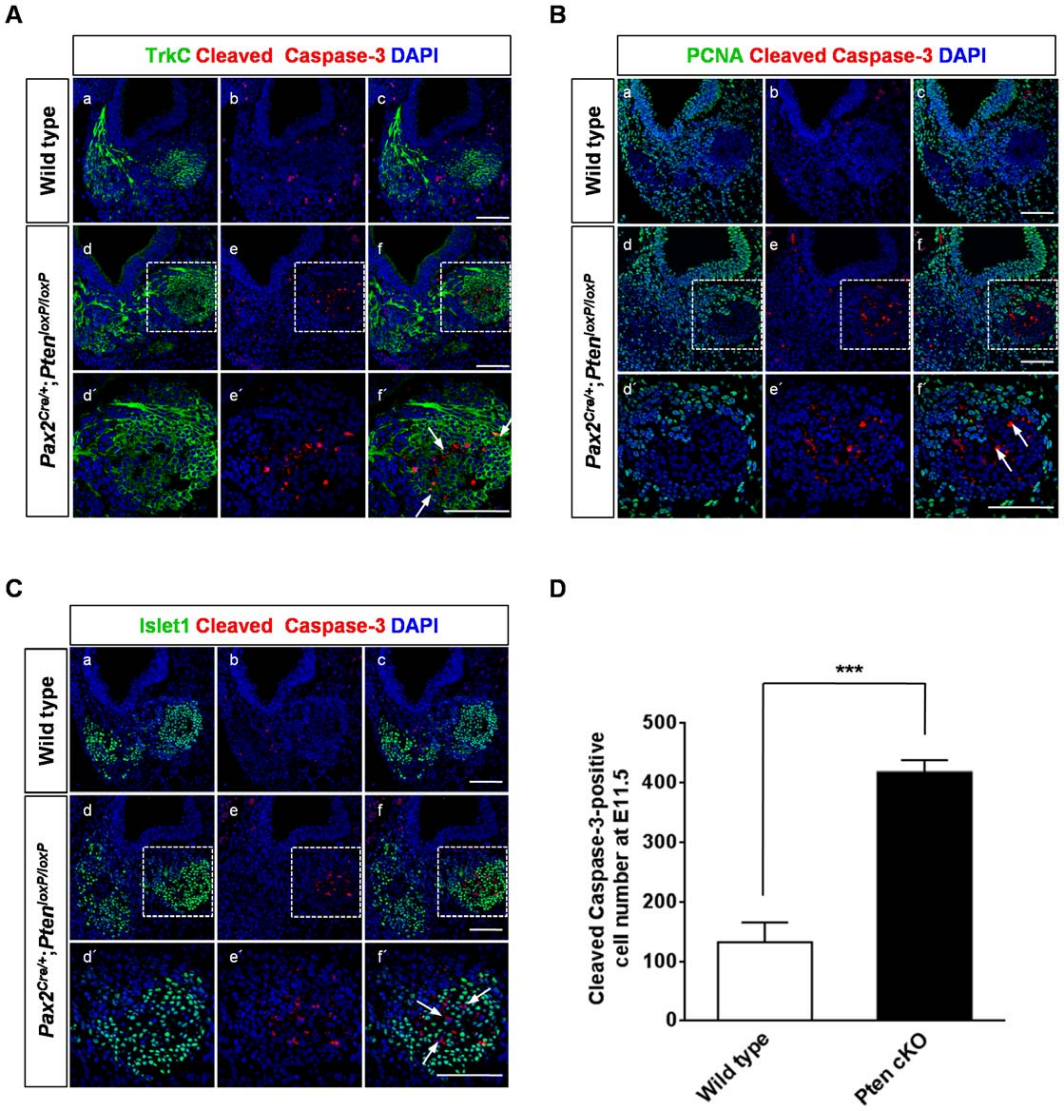
Neuronal apoptosis in the cochleovestibular ganglion (CVG) complex of *Pten*-deficient mice. (A–D) Apoptotic cells in the CVG were stained with anti-cleaved caspase-3 antibody at E11.5. Cleaved caspase-3 immunoreactivity (red) increased substantially in *Pten*-deficient mice compared to that in wild-type mice. (A) Apoptotic neurons were occasionally co-localized with TrkC-positive (green) (arrows in f) or negative cells. Higher magnification images of d, é, and f are shown in the insets in d, e, and f, respectively. Scale bars: 100 µm. (B) Proliferating PCNA-positive cells (green) were not seen in apoptotic neurons (arrows in f*** ´***). Higher magnification images of d, e, and f are shown in the insets in d, e, and f, respectively. Scale bars: 100 µm. (C) Cleaved caspase-3-positive cells (red) were also Islet1-negative cells (arrows in f*** ´***). Apoptotic neurons (red) were distributed in the core of the non-proliferative area that expressed Islet1 (green). Higher magnification images of d, e, and f are shown in the insets in d, e, and f, respectively. Scale bars: 100 µm. (D) Numbers of cleaved caspase-3-positive apoptotic cells in the CVG were significantly increased in *Pten*-deficient mice at E11.5 (3 cochleae, *P<*0.001).

**Figure 6:**
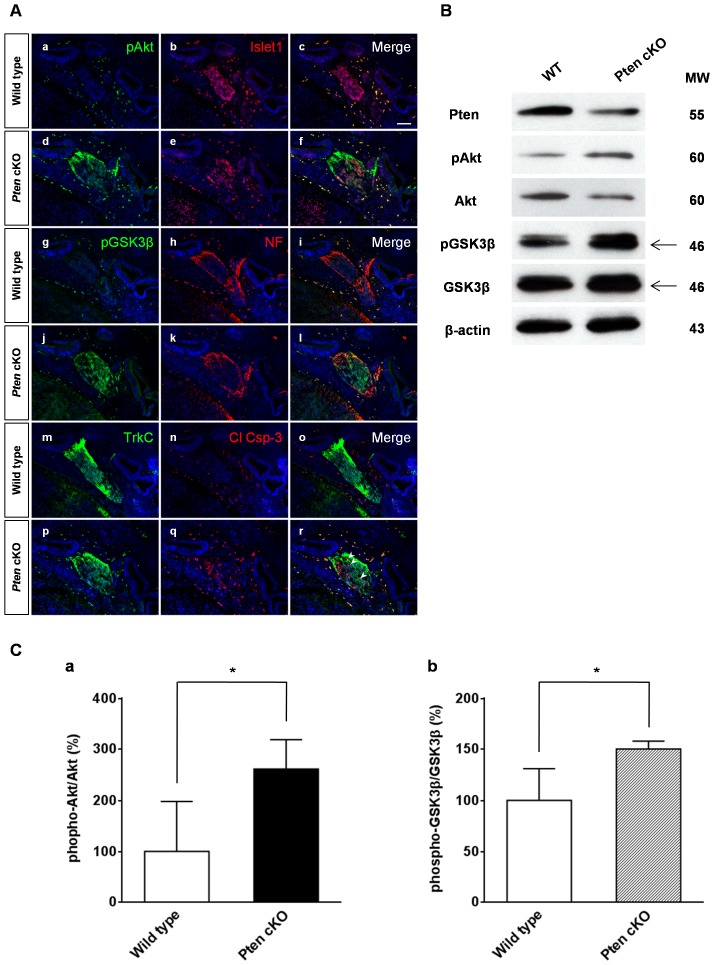
Akt and GSK3β phosphorylation in *Pten*-deficient mice. (A) Phosphorylation of Akt and GSK3β was measured by immunofluorescence staining using anti-phospho-Akt (Ser473) (pAkt) and anti-phospho-GSK3β (Ser9) (pGSK3β) antibodies at E13.5. Compared to wild-type spiral ganglia, Akt phosphorylation (green) increased substantially in *Pten*-deficient mice (a–f). GSK3β phosphorylation (green) was also highly expressed in the spiral ganglia of *Pten*-deficient mice compared to that in wild-type mice (g–l). In the absence of *Pten*, all pAkt and pGSK3β cells were maintained in Islet-1 positive cells (red) (a–l). Wild-type mice showed well-organized patterns of Islet-1-positive cells (a–c), whereas *Pten*-deficient mice showed a slightly scattered form of spiral ganglia (d–f). Cleaved caspase-3-positive apoptotic neurons (red) were sometimes co-localized with TrkC-positive cells (green) (arrowheads in r). DAPI-stained nuclei (blue) are seen in all images. Scale bar: 100 µm. (B) Western blotting analysis of Pten, pAkt, Akt, pGSK3β, GSK3β, and β-actin expression in the inner ear at E14.5. Proteins were extracted from four pooled inner ears at E14.5. (C) The relative intensity of each phospho-protein was normalized to the total level of the same protein. Levels of both pAkt (a) and pGSK3β (b) were significantly increased in *Pten* cKO mice (4 cochleae, *P<*0.05).

We examined the neuronal markers NeuroD and Islet1 at E10.5 to understand the effect of *Pten* deletion on the CVG (Fig. 4). Although the *Pten* cKO NeuroD-positive area was not reduced compared to that of the control, the number of NeuroD-positive cells was decreased by 40% in the CVG (5 cochleae, *P*<0.001; Fig. 4B). This reduction was already evident in the epithelium before delamination of neuroblasts. The number of NeuroD-positive cells within the epithelium was reduced by 23% in *Pten* cKO mice compared to wild-type mice (8 cochleae, *P*<0.05; Fig. 4C). Although NeuroD-positive CVG cells were disorganized and the neuroblasts became flattened in *Pten* cKO mice, they were still co-localized with PCNA-positive cells, as observed in the wild-type (data not shown). At neuronal maturation after exit from the cell cycle, neurons appeared to be expressed by both Islet1-positive and NeuroD-negative cells in the CVG at E10.5 (arrows in Fig. 4A, e and f). Islet1-positive and NeuroD-negative neurons also decreased significantly compared to those in the wild-type (6 cochleae, *P*<0.05; Fig. 4D). Furthermore, apoptosis in the spiral ganglion increased markedly from E11.5 in *Pten*-deficient mice compared to that in the wild-type (Figs. 5 and 6). To identify whether the cleaved caspase-3-positive cells were neurons or Schwann cells, we determined the co-localization of apoptotic cells using immunoreactivity to Tuj1, a neuronal marker, and Sox10, a Schwann cell marker (Fig. S5). Immunofluorescence results showed that cleaved caspase-3 signals were co-localized in neurons expressed with Tuj1-positive cells but not with Sox10-positive Schwann cells (arrows in Fig. S5, B*** ´***).

We performed PCNA immunofluorescence at E11.5 to examine whether the apoptotic neuronal cells were in a differentiation stage (Fig. 5B). Apoptotic neurons were distributed in the core of the non-proliferative area (arrows in Fig. 5B, f) and expressed Islet1, a neuronal marker in late blasts and immature neural precursors (arrows in Fig. 5C, f). Numbers of cleaved caspase-3-positive apoptotic cells at E11.5 were increased by 215% in Pten cKO mice compared to wild-type mice (3 cochleae, *P<0.001*; Fig. 5D). These results indicate that apoptotic neurons are not proliferating but rather are differentiating cells.

### Signaling Pathway

We investigated Akt activity to determine the mechanisms of the *Pten* loss-induced spiral ganglion neuronal phenotype, as it is well known that PTEN loss promotes Akt signaling pathway activity. Akt activation was assessed by detecting Akt phosphorylation at the Ser473 residue. As shown in Figure 6, Akt phosphorylation increased in the spiral ganglion of *Pten*-deficient mice compared to that of wild-type mice (Fig. 6A, a–f). The relative intensity of the phospho-Akt signal was increased by 162% in *Pten*-deficient mice compared to wild-type mice (4 cochleae, *P*<0.05; Fig. 6B and C, a). We observed Ser9-phosphorylation of the GSK3β protein in the Pten-deleted inner ear at E13.5, the neuronal differentiation stage, which was co-expressed in the phospho-Akt-positive cells of the spiral ganglion (Fig. 6A, d and j). The level of GSK3β phosphorylation at E13.5 was sustained at E14.5 (data not shown). However, at an earlier stage, such as E11.5, GSK3β phosphorylation was detected in both wild-type and Pten-deleted mice (data not shown). The r*elative intensity of the phospho-GSK3β/GSK signal was increased by ∼50%* in *Pten*-deleted mice compared to wild-type mice (4 cochleae, *P*<0.05; Fig. 6B and C, b). The cleaved caspase-3-positive cells no longer expressed phospho-Akt, phospho-GSK3β, or Islet1.

## Discussion

In this study, we explored whether *Pten* plays a neuronal role in the regulation of development in the mouse inner ear. We found that inner ear-specific *Pten* deficiency resulted in severe neuronal abnormalities such as apoptosis of spiral ganglion with disorganization of nerve innervations. This is the first report of on the novel functions of *Pten* in regulating neuronal survival, differentiation, and axon pathfinding of neurons in the developing inner ear.

### *Pten* is Required for the Morphogenesis of the Developing Inner Ear

Our results provide an initial characterization of the *Pten*-depleted inner ear. Notably, *Pten*-deficient mice have enlargement of the entire membranous labyrinth, which is dramatic in the endolymphatic sac (Fig. 2A, B). This phenotype is similar to that in the inner ear of anion-exchanger *Pendrin*-knockout mice [36]. This dysmorphogenesis may be associated with changes in ion channels, including transporters and exchangers. Recent studies have shown that *Pten* regulates ion channels through PI3K/Akt signaling [37], [38], [39]. The molecular mechanism behind the morphological defects in the *Pten*-depleted inner ear, however, requires further study.

Specifically, we observed neuronal defects in the *Pten*-deficient inner ear (Fig. 3), leading to aberrant axon pathfinding of spiral ganglia and disruptions of the accurate connections to the hair cells. This abnormality was influenced by the substantial reduction of neuronal cell number after neural fate commitment of progenitors (Figs. 3 and 4). The disorganized radial fibers and neuronal loss in the inner ear of *Pten* cKO mice is similar to the phenotypes of several mutants (*e.g*., *Brn3a* ^−/−^, *TrkC* ^−/−^, or *ErbB2* ^−/−^ required for the differentiation of the spiral ganglia [40], [41]) and suggests that *Pten* may play a role in these gene*-*mediated signaling pathway. Thus, our results suggest that *Pten* is a functional regulator involved in axon pathfinding of the spiral ganglia during inner ear development.

### *Pten* Deficiency Induced Apoptosis of Differentiated Spiral Ganglia

Several studies on *Pten*-deficient mice have reported hypertrophic characteristics such as an enlarged brain and tumorigenesis in various organs [20], [21], [22], [23]. In contrast, we found that *Pten* deficiency induced apoptosis of pre-differentiated cells but not in proliferating CVG in the developing inner ear (Fig. 5). We observed neuronal loss of spiral ganglia in *Neurog1^Cre/+^*;*Pten^loxP/loxP^* mice (Fig. 3B), similar to *Pax2^Cre/+^*;*Pten^loxP/loxP^* mice (Fig. 3A). This contradiction suggests that *Pten* has a particular function in the development of cochlear ganglia. The role of *Pten* in the inner ear agrees with a study in which *PTEN*-antisense in a neuronal cell line induced cell death with a loss of differentiating cells [26]. These results indicate that *Pten* deficiency promotes apoptosis in differentiating neurons in the delaminated CVG. A previous study reported that prolonged PI3K activation could trigger apoptosis via chronic induction of a cell-cycle component [42]. Thus, further experiments are required to determine the mechanism by which *Pten* deficiency induces apoptosis in spiral ganglia.

### *Pten* Deficiency Causes GSK3β Phosphorylation in Spiral Ganglion Differentiation

To verify the proposed signaling mechanisms for spiral ganglion defects in *Pax2^Cre/+^*;*Pten^loxP/loxP^* mice, we examined whether *Pten* deficiency (Fig. S6) upregulates Ser473-phosphorylation of Akt and triggers Ser9-phosphorylation of GSK3β (pGSK3β) in the PI3K signaling cascade. As shown in Figure 6, *w*e found hyperactive Akt and hypoactive GSK3β by immunofluorescence in the spiral ganglion of Pten-deleted inner ears at E13.5. At E11.5, the level of pGSK3β in *Pten* cKO mice was similar to that of wild-type mice (data not shown). *Although* wild-type mice exhibited a decreased level of pGSK3β, indicating increased activation at E13.5, Pten cKO mice did not decrease the GSK3β phosphorylation level at the same developmental stage (Fig. 6). *It was recently* established that precise regulation of GSK3 activity is required to control neuronal differentiation. Inhibiting GSK3 facilitates or prevents axon formation and extension by regulating phosphorylation of its substrates; *i*.*e*., microtubule-associated protein 1B, collapsin response mediator protein 2, or adenomatosis polyposis coli [43], [44]. Dysregulation of GSK3β in spiral ganglion neurons may affect the neuronal phenotype of *Pten*-deficient mice by modulating its substrates, suggesting that it acts to inhibit axonal growth in *Pten* cKO mice. Further studies are required to determine whether the *Pten*-deficiency-induced neuronal defect is attributable to GSK3β inactivation in spiral ganglia. GSK3 plays a role in coordinating various developmental signaling pathways, including Wnt, sonic hedgehog, fibroblast growth factor, and the Notch signaling pathways [16], [45], [46], [47]. Since GSK3β is not well controlled in *Pten* cKO mice, the interaction with those signaling networks may be affected during normal development of cochlear ganglia. Our results suggest that GSK3β is a regulator of neurogenesis that also maintains spiral ganglia. Thus, the results presented here provide new insights into the role of Pten/Akt/GSK3β signaling during neuronal differentiation of the developing inner ear.

In conclusion, *Pten* loss-induced neuronal defects reveal the novel role of *Pten* in the auditory ganglia during inner ear development. *Pten* is essential for the survival of spiral ganglia after neural fate specification and play a role in the accurate axon pathfinding of spiral ganglia to hair cells in the organ of Corti. Further investigations on the molecular mechanisms of *Pten* function will increase our knowledge of sensory neural development in the inner ear.

## Supporting Information

Figure S1 **Neuronal Pten expression during inner ear development in wild-type mice.** (A) In the neurons at E10.5, E14.5, E16.5, and E18.5, the expression of Pten (red) partly overlapped with that of neurofilament (green), which was expressed in both the neuronal cell body and neuritis (arrows in b–d, f–h, j–l, and n–p). Higher magnification images of boxed regions in a–p are shown in á–p, respectively. Scale bars: 100 µm in a–p; 20 µm in á–p. (B) In the vestibule at E16.5, Pten expression (red) was detected in the MyoVIIa-positive sensory epithelium (green) (arrows in b–d), the non-sensory epithelium (arrowheads in c and d), neurofilament-positive neurites (green) (arrowheads in f–h), and vestibular ganglia (green) (arrows in j–l). Higher magnification images of boxed regions in a–l are shown in á–l, respectively. c, crista; s, saccule; u, utricle. Scale bars: 100 µm in a–l; 50 µm in á–h; 10 µm in í–l.
(TIF)

Figure S2 **Confocal images of the hair bundles of the organ of Corti at E18.5.** (A–C) Stereociliary bundle (green) and kinocilia (red) labeling of the surface of the organ of Corti from the basal turn showed the regular pattern of a single row of inner hair cells and three rows of outer hair cells. Scale bars: 50 µm in A–C; 10 µm in Á–Ć. (D–F) In *Pax2^Cre/+^*;*Pten^loxP/loxP^* mice, some variations in the position and orientation of hair bundles were observed in outer hair cells and inner hair cells, which were obvious in the region with increased numbers of hair cells (arrows in F). Scale bars: 50 µm in D–F; 10 µm in D–F.
(TIF)

Figure S3 **Neuronal loss of vestibular ganglia in** ***Pten*** **-deficient mice at E16.5.** (A) Tuj1 immunoreactivity (green) were reduced in the vestibular ganglion of *Pax2^Cre/+^*;*Pten^loxP/loxP^* mice. U, utricle; VG, vestibular ganglion. Scale bar: 100 µm. (B) Numbers of vestibular ganglia were significantly reduced compared to wild-type mice (7 cochleae, *P*<0.01).
(TIF)

Figure S4 **Epithelial phenotype in** ***Neurog1^Cre/+^*** **;** ***Pten^loxP/loxP^*** **and** ***Atoh1^Cre/+^*** **;** ***Pten^loxP/loxP^*** **mice.** The morphological pattern of the epithelium was revealed by whole-mount phalloidin (green) with p75^NTR^ (red) immunofluorescence. (A) At E18.5, normally organized cochlear hair cells were seen in *Neurog1^Cre/+^*;*Pten^loxP/loxP^* mice injected with tamoxifen between E8.5 and E11.5 (a, b). Scale bar: 20 µm. (B) Tamoxifen-inducible *Pten* deletion from E13.5 to E14.5 in *Atoh1^Cre/+^*;*Pten^loxP/loxP^* mice included three rows of outer and one row of inner hair cells compared to that in wild-type mice (a, b). Scale bar: 20 µm.
(TIF)

Figure S5 **Apoptotic neurons in the cochleovestibular ganglion (CVG) complex of** ***Pax2^Cre/+^*** **;** ***Pten^loxP/loxP^*** **embryos.** (A–D*** ´***) Cleaved caspase-3-positive apoptotic cells (red) in the CVG were stained with Tuj1 (green), a neuronal marker, or Sox10 (green), a Schwann cell marker, at E12.5. (B, B*** ´***) Apoptotic cells were co-localized with Tuj1-positive neurons in the CVG (arrows in B*** ´***). (D, D*** ´***) In contrast, Sox10-positive Schwann cells did not stain with cleaved caspase-3 antibody. Scale bars: 100 µm.
(TIF)

Figure S6 **Reduction in Pten-positive immunoreactivity in** ***Pax2^Cre/+^*** **;** ***Pten^loxP/loxP^*** **mice.** Pten immunopositivity (red) in the spiral ganglion at E16.5 was significantly decreased in *Pax2^Cre/+^*;*Pten^loxP/loxP^* mice compared to wild-type mice. DAPI-stained nuclei (blue) are seen in all images. SG, spiral ganglion. Scale bar: 100 µm
(TIF)
