# Supplementary figures and images for: Conditional Deletion of Pten Leads to Defects in Nerve Innervation and Neuronal Survival in Inner Ear Development
Source: PLoS One. 2013 Feb 5;8(2):e55609. doi: 10.1371/journal.pone.0055609 (PMC3564925; doi:10.1371/journal.pone.0055609)

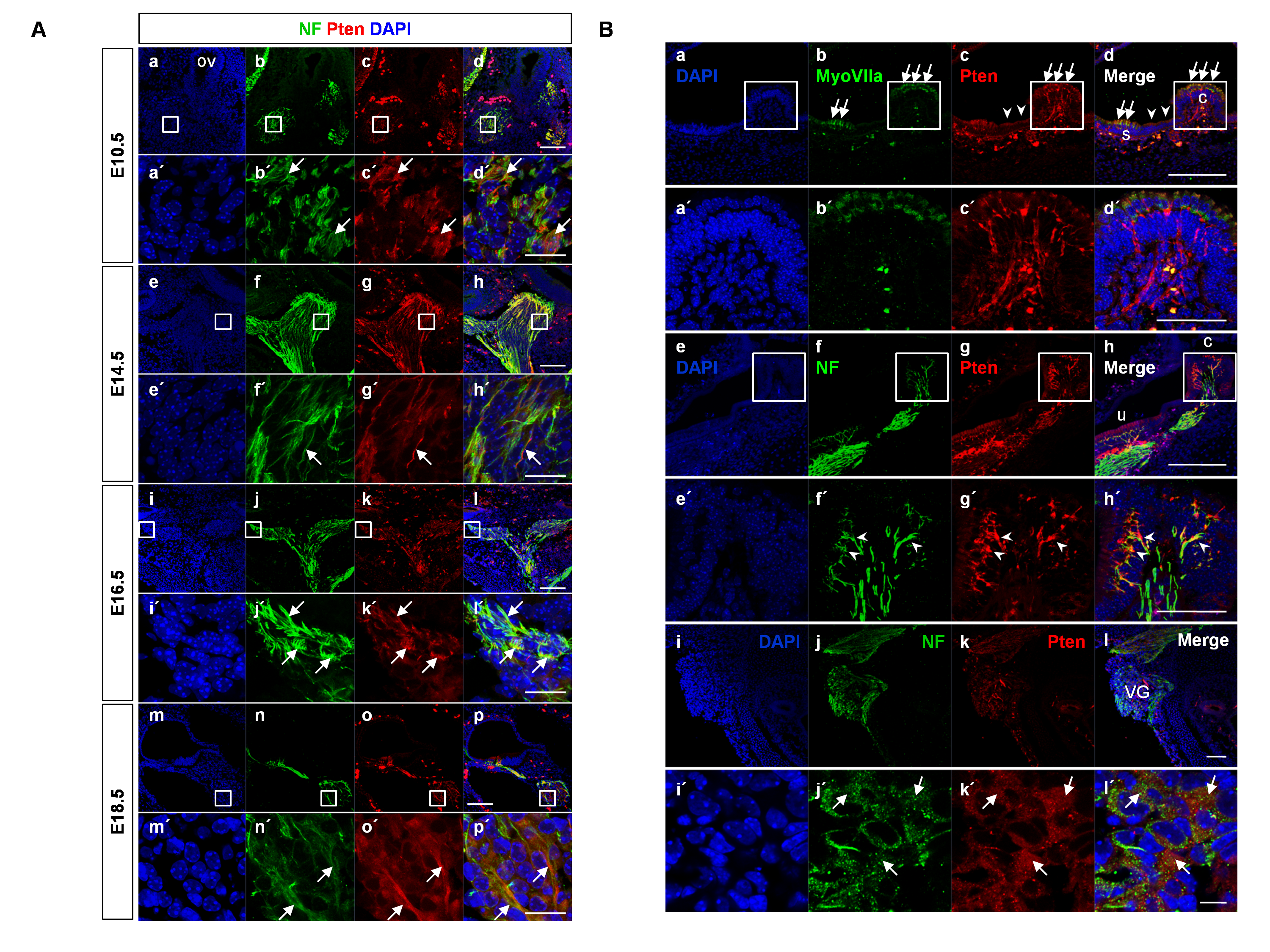

Supplement: Figure S1 — Neuronal Pten expression during inner ear development in wild-type mice. (A) In the neurons at E10.5, E14.5, E16.5, and E18.5, the expression of Pten (red) partly overlapped with that of neurofilament (green), which was expressed in both the neuronal cell body and neuritis (arrows in b–d, f–h, j–l, and n–p). Higher magnification images of boxed regions in a–p are shown in á–p, respectively. Scale bars: 100 µm in a–p; 20 µm in á–p. (B) In the vestibule at E16.5, Pten expression (red) was detected in the MyoVIIa-positive sensory epithelium (green) (arrows in b–d), the non-sensory epithelium (arrowheads in c and d), neurofilament-positive neurites (green) (arrowheads in f–h), and vestibular ganglia (green) (arrows in j–l). Higher magnification images of boxed regions in a–l are shown in á–l, respectively. c, crista; s, saccule; u, utricle. Scale bars: 100 µm in a–l; 50 µm in á–h; 10 µm in í–l. (TIF) [file pone.0055609.s001.tif]

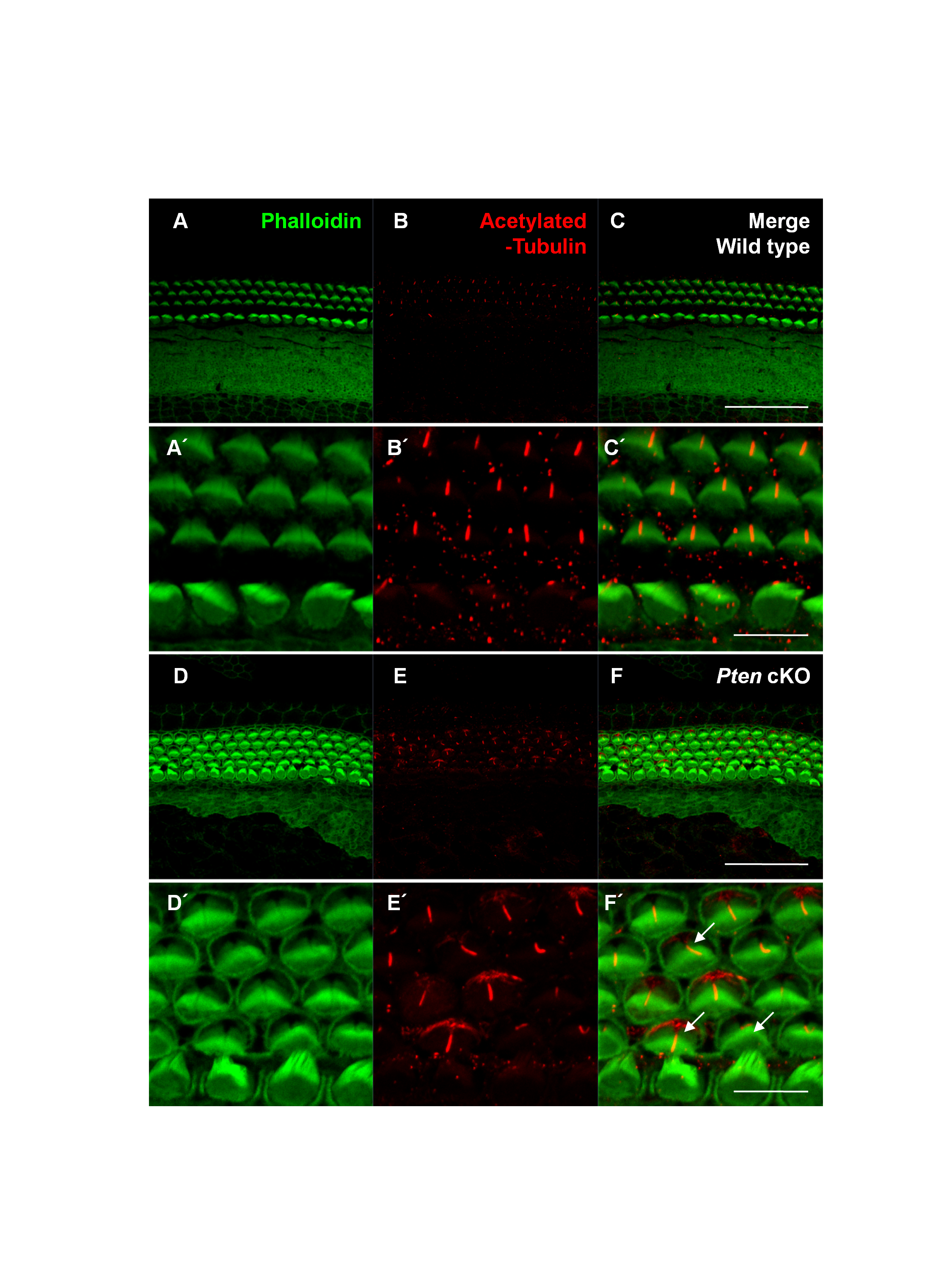

Supplement: Figure S2 — Confocal images of the hair bundles of the organ of Corti at E18.5. (A–C) Stereociliary bundle (green) and kinocilia (red) labeling of the surface of the organ of Corti from the basal turn showed the regular pattern of a single row of inner hair cells and three rows of outer hair cells. Scale bars: 50 µm in A–C; 10 µm in Á–Ć. (D–F) In Pax2Cre/+;PtenloxP/loxP mice, some variations in the position and orientation of hair bundles were observed in outer hair cells and inner hair cells, which were obvious in the region with increased numbers of hair cells (arrows in F). Scale bars: 50 µm in D–F; 10 µm in D–F. (TIF) [file pone.0055609.s002.tif]

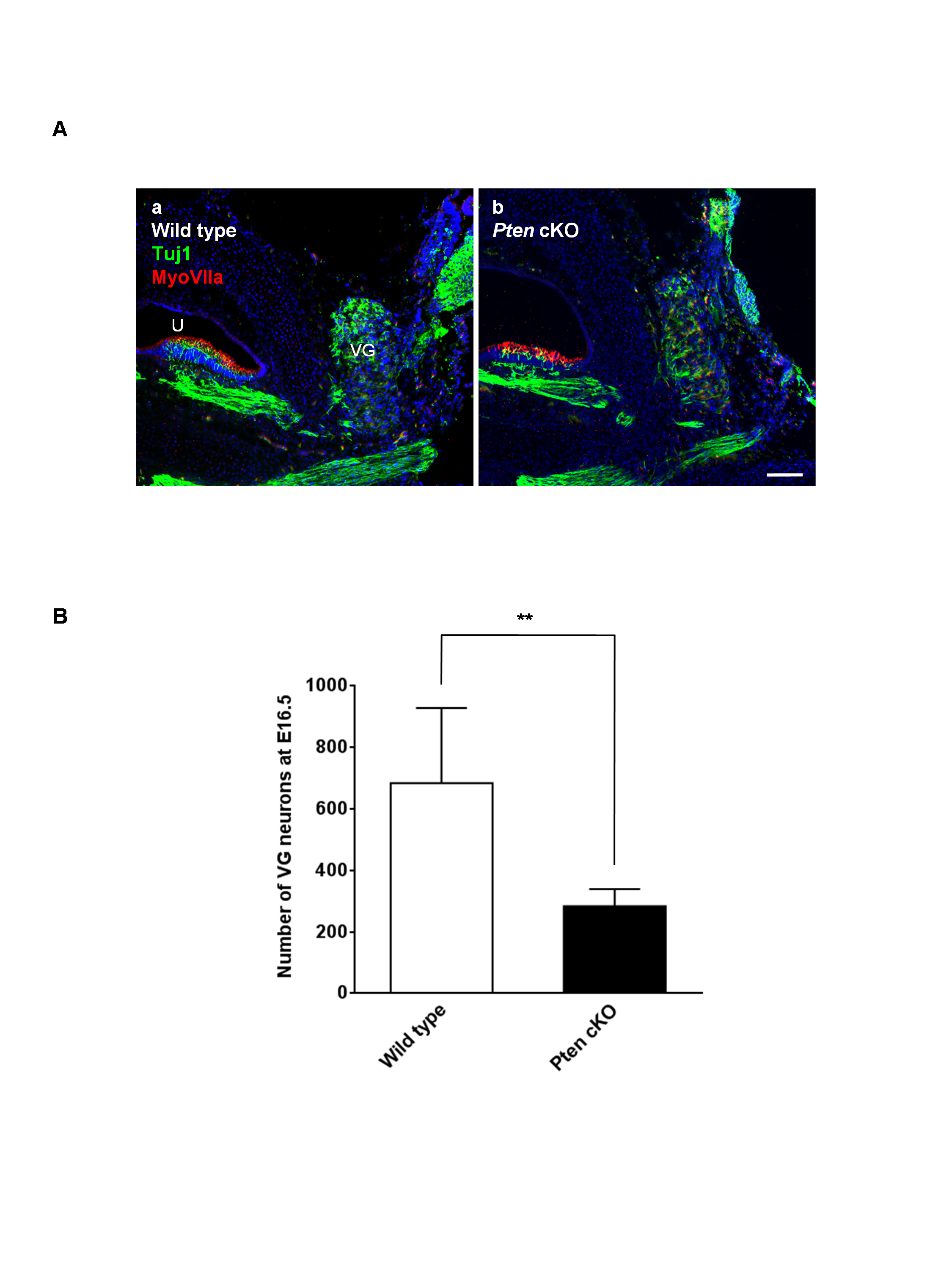

Supplement: Figure S3 — Neuronal loss of vestibular ganglia in Pten -deficient mice at E16.5. (A) Tuj1 immunoreactivity (green) were reduced in the vestibular ganglion of Pax2Cre/+;PtenloxP/loxP mice. U, utricle; VG, vestibular ganglion. Scale bar: 100 µm. (B) Numbers of vestibular ganglia were significantly reduced compared to wild-type mice (7 cochleae, P<0.01). (TIF) [file pone.0055609.s003.tif]

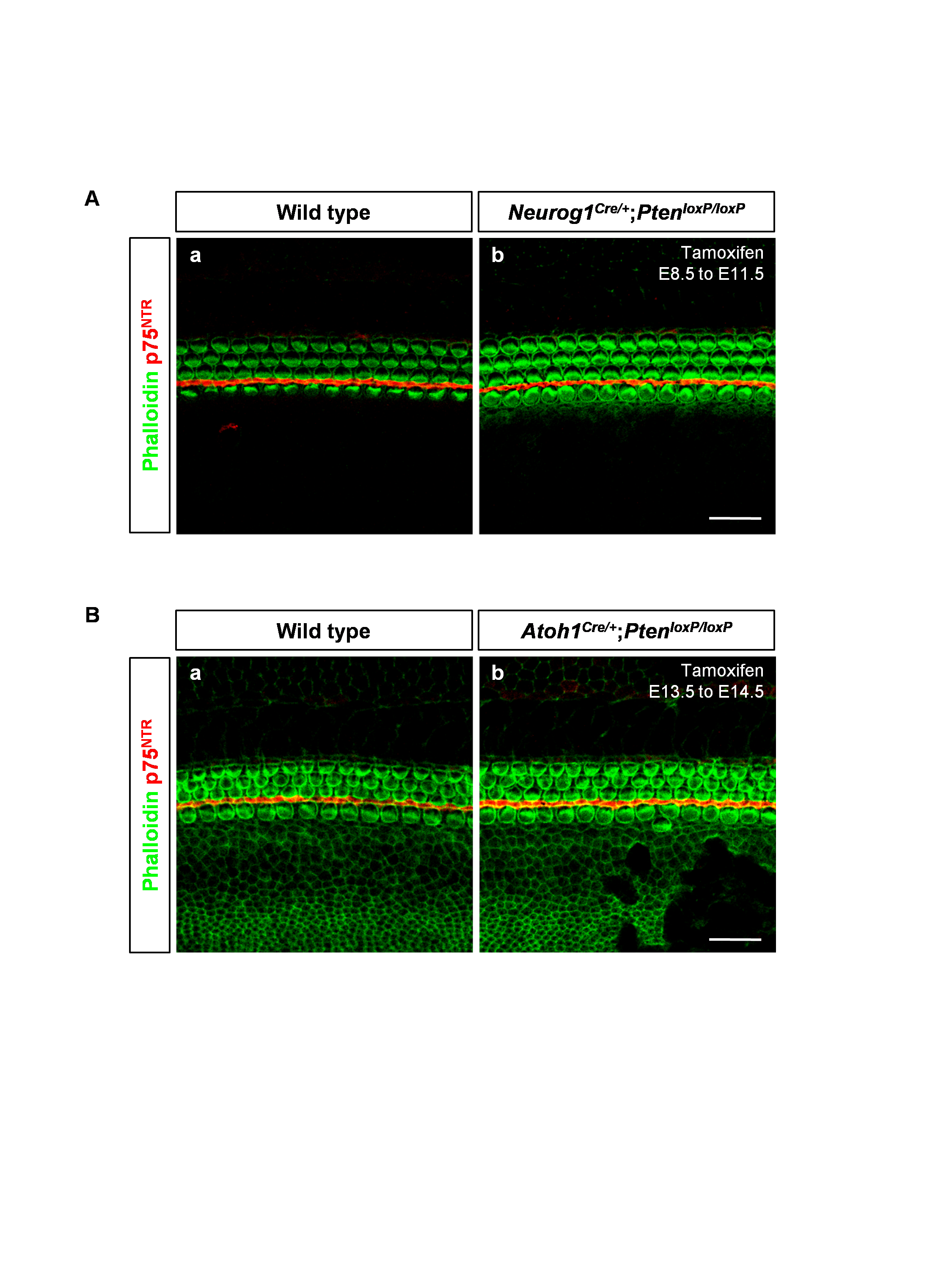

Supplement: Figure S4 — Epithelial phenotype in Neurog1Cre/+ ; PtenloxP/loxP and Atoh1Cre/+ ; PtenloxP/loxP mice. The morphological pattern of the epithelium was revealed by whole-mount phalloidin (green) with p75NTR (red) immunofluorescence. (A) At E18.5, normally organized cochlear hair cells were seen in Neurog1Cre/+;PtenloxP/loxP mice injected with tamoxifen between E8.5 and E11.5 (a, b). Scale bar: 20 µm. (B) Tamoxifen-inducible Pten deletion from E13.5 to E14.5 in Atoh1Cre/+;PtenloxP/loxP mice included three rows of outer and one row of inner hair cells compared to that in wild-type mice (a, b). Scale bar: 20 µm. (TIF) [file pone.0055609.s004.tif]

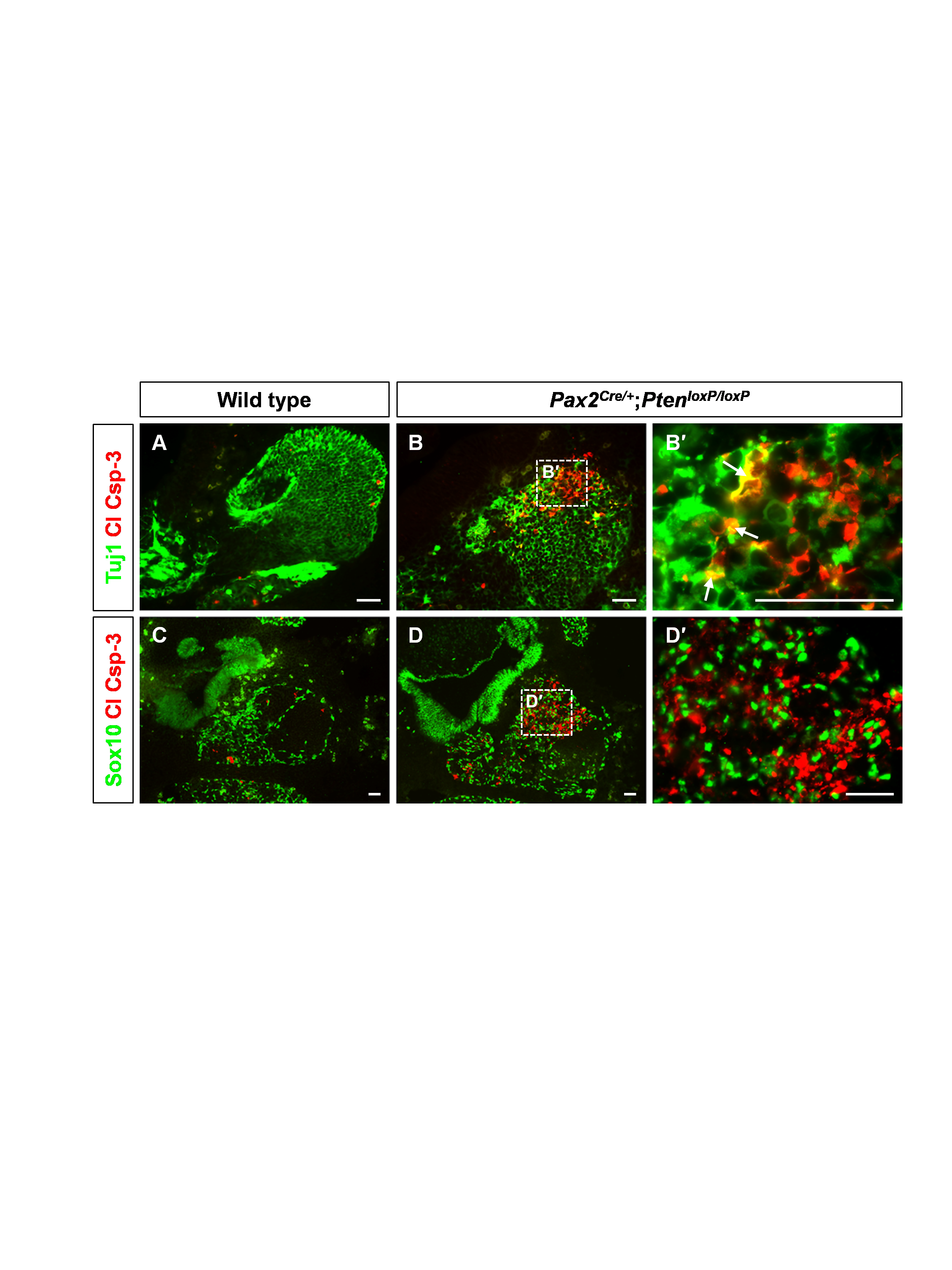

Supplement: Figure S5 — Apoptotic neurons in the cochleovestibular ganglion (CVG) complex of Pax2Cre/+ ; PtenloxP/loxP embryos. (A–D ´) Cleaved caspase-3-positive apoptotic cells (red) in the CVG were stained with Tuj1 (green), a neuronal marker, or Sox10 (green), a Schwann cell marker, at E12.5. (B, B ´) Apoptotic cells were co-localized with Tuj1-positive neurons in the CVG (arrows in B ´). (D, D ´) In contrast, Sox10-positive Schwann cells did not stain with cleaved caspase-3 antibody. Scale bars: 100 µm. (TIF) [file pone.0055609.s005.tif]

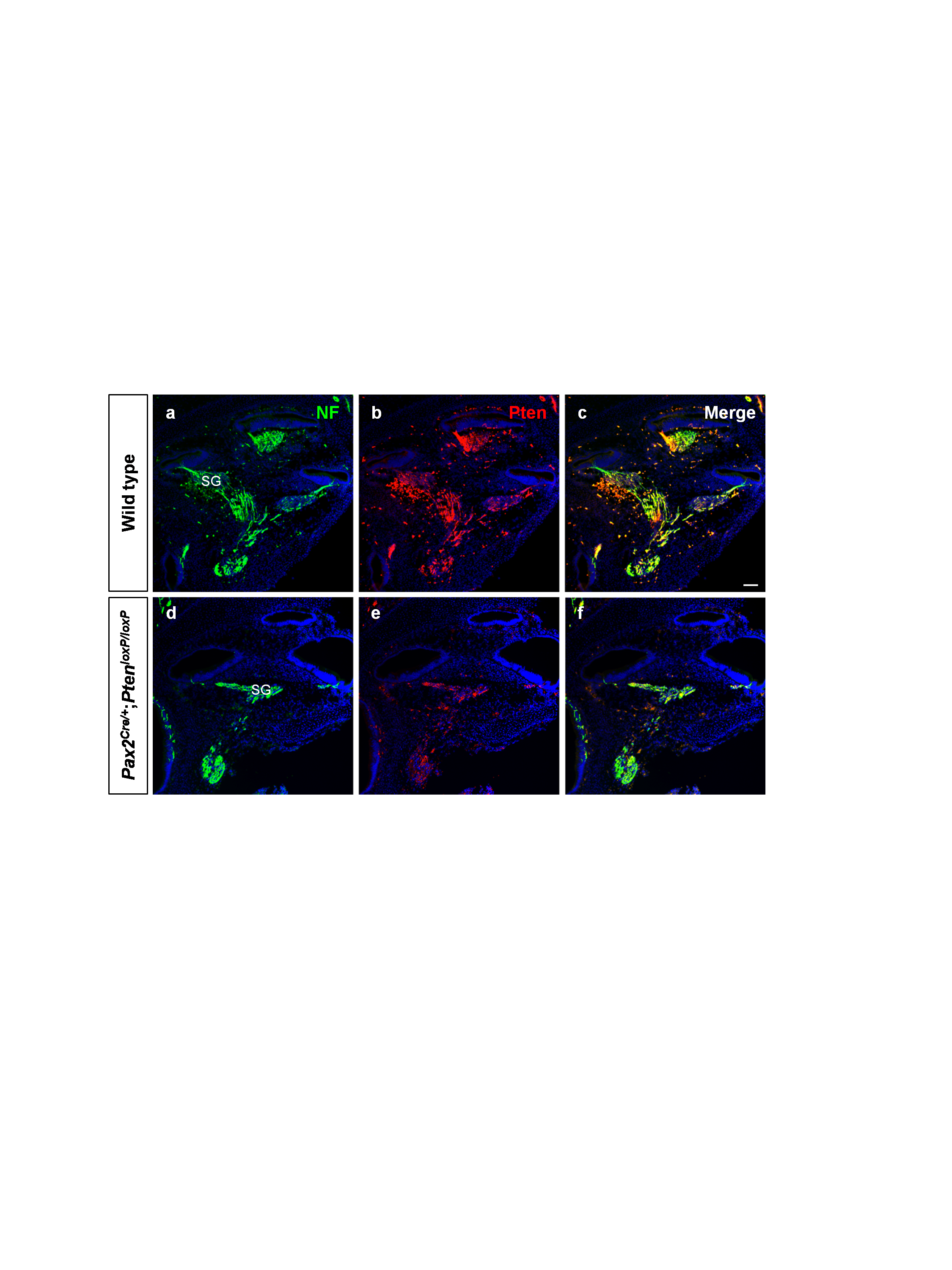

Supplement: Figure S6 — Reduction in Pten-positive immunoreactivity in Pax2Cre/+ ; PtenloxP/loxP mice. Pten immunopositivity (red) in the spiral ganglion at E16.5 was significantly decreased in Pax2Cre/+;PtenloxP/loxP mice compared to wild-type mice. DAPI-stained nuclei (blue) are seen in all images. SG, spiral ganglion. Scale bar: 100 µm (TIF) [file pone.0055609.s006.tif]
